# The potential anti-cancer effects of quercetin on blood, prostate and lung cancers: An update

**DOI:** 10.3389/fimmu.2023.1077531

**Published:** 2023-02-28

**Authors:** Noushin Lotfi, Zahra Yousefi, Marjan Golabi, Parvin Khalilian, Behrooz Ghezelbash, Mina Montazeri, Mohammad Hossein Shams, Parnian Zolfaghari Baghbadorani, Nahid Eskandari

**Affiliations:** ^1^ Department of Medical Immunology, School of Medicine, Isfahan University of Medical Sciences, Isfahan, Iran; ^2^ School of Allied Medical Sciences, Shahroud University of Medical Sciences, Shahroud, Iran; ^3^ Department of Medical Immunology, School of Medicine, Lorestan University of Medical Sciences, Khorramabad, Iran

**Keywords:** quercetin, apoptosis, therapy, blood cancer, lung cancer, prostate cancer

## Abstract

Cancer is caused by abnormal proliferation of cells and aberrant recognition of the immune system. According to recent studies, natural products are most likely to be effective at preventing cancer without causing any noticeable complications. Among the bioactive flavonoids found in fruits and vegetables, quercetin is known for its anti-inflammatory, antioxidant, and anticancer properties. This review aims to highlight the potential therapeutic effects of quercetin on some different types of cancers including blood, lung and prostate cancers.

## Introduction

One of the important leading cause of death in the worldwide is cancer, followed by heart disease. Each year, the numbers of new patients with various cancers as well as the death rate from different types of malignancies both increased. Therefore, development of novel and effective therapeutic approaches is required to management of cancer progression, improvement of clinical manifections, and effective treatment of this disorder (1).

Among the various types of malignancies, blood, prostate, and lung cancers have been identified as the most prevalent forms of tumors with the highest incidence and death worldwide, particularly in the United States (US) (2). Blood cancers including leukemia, lymphoma, and myeloma account for approximately 10% of all diagnosed tumors with about 5 years survival rate. Blood cancers have effect on function and production of blood cells. There are numerous different methods for treating blood malignancies. Hematological malignancies are mostly treated with chemotherapy and systemic anti-cancer medications. However, the survival rate of patients with various blood malignancies is low due to the ineffectiveness of current medications and the high likelihood of relapse after treatment (2, 3). Therefore, identification of more comprehensive and functional treatments will be given more attention.

With significantly varying incidence and mortality data, lung cancer has also been highlighted as the primary reason for cancer-related deaths worldwide (4). In 2022 nearly 350 deaths per day were reported in the US from this solid tumor. Lung cancer affects over 2.8 million people worldwide (2, 5). Lung cancer and breast cancer account for about one-third of all diagnosed cases in female. Two important subtypes of this tumor are small cell lung cancer and non-small cell lung cancer. Depending on type and stage of the disease at the time of diagnosis, lung cancer patients treated with various approaches including immunotherapy, chemotherapy, and surgery. These treatments improved the relative survival rates of patients over time. However, two years survival rates estimated for lung cancer patients. Early detection and improvement of therapeutic approaches could significantly reduce lung cancer mortality (4, 6).

Prostate cancer is the leading cause of death among men (7). Also, it is the second most common cause of cancer-related death in the US, after lung cancer (8). The disease mainly affects on older men with an average age of about 66 years. Abnormal division of prostate cells and abnormal growth of the prostate lead to this disease. Similar to other cancers, treatment methods on prostate cancer treatment revealed many side effects. Given that increase the incidence rate of men with prostate cancer, it is crucial to find new strategies for managing or treating of this disease. Today, some dietary recommendations have been made to treat various conditions like cancer, such as nutrition and leading a better lifestyle, but the results and advice are not yet useful (9, 10).

Fruits and vegetables include the flavonoid subgroup of flavanols known as quercetin (Que; 3,5,7,3,4’-pentahydroxy flavonoids). This substance has numerous biological effects including anti-inflammatory, anti-cancer, and antioxidant effects. In addition, it revealed anti-cancer effects on cancer cells by regulating a number of molecular factors involved in signaling pathways, such as PI3K/Akt/mTOR, Wnt/β-catenin, and MAPK/ERK1. Quercetin increases apoptosis and autophagy in cancer by activating caspase-3, inhibiting the phosphorylation of Akt, mTOR, and ERK, lessening β-catenin, and stabilizing the stabilization of HIF-1α. In addition, by reducing MMP and VEGF secretion, it inhibits cancer cell metastasis. Additionally, this compound leads to cancer cells apoptosis by decreasing bioenergy and targeting mitochondria (11).

The antitumor efficacy of quercetin derivatives varied from quercetin based on the subunit location and chain length. Insertion of Phenolic hydroxyl group such as etherification (O-alkylation) cancer cell proliferation may effectively suppressed. Modified quercetin properties vary in different cancer cell lines and additional researches is required to identify modified quercetin properties (12–14). Additionally, several nanoparticles placed onto the surface of the quercetin, including chemotherapeutic medicines like mitoxantrone (MTX) and adriamycin (ADR). This modified quercetin may reduce chemotherapeutic medication side effects and multi-drug resistance while enhancing synergistic anticancer benefits (15).

Regarding that blood, lung, and prostate cancers accounting for almost one-half of all diagnosed cases with tumor as well as different reports of therapeutic effects of quercetin in cancer studies, this study aimed to collect information and review all measures taken. Therefore, by recognizing the gaps in essays related to this drug and providing suggestions and solutions, it may be possible to use it more effectively to treat these cancers in the future.

## Anti-angiogenesis effect of quercetin on cancer

Angiogenesis, which produces all capillaries, is regulated by a number of substances, including endostatin, adhesion molecules, and growth factors. Angiogenesis is essential for the growth of reproductive systems and the repair of injuries. Dysregulated angiogenesis contributes to tumor growth and metastasis. The tumor angiogenesis depends greatly on interactions between tumor cells and endothelial cells. VEGF play an important role in the growth and survival of endothelial cells, which promotes cell proliferation, increases vascular permeability, extravasates plasma fibrin, deposits cellulose, and tumor angiogenesis (16).

Natural products are a key component of cancer prevention. Many phytochemicals could be used to develop antiangiogenic medications. Flavonoids are polyphenolic substances that are present in nearly all food plants and have antiviral, antibacterial, anti-inflammatory, and cytoprotective effects on several animal and human cell types. According to epidemiological studies, high consumption of flavonoids may reduce cancer risks (17).

Quercetin have revealed an anti-tumor effect by reducing development of blood vessels. In addition, this natural component decrease tumor growth through targeting VEGFR-2-mediated angiogenesis pathway and suppressing the downstream regulatory component AKT in prostate and breast malignancies. The potential of quercetin to inhibit angiogenesis in drug-resistant cells increases its effects on anti-cancer medicines (18).

## Anti-metastatic effect of quercetin on cancer

Metastasis is known as a major cause of cancer progression (19). In this process, cancer cells escape from the attack of the immune system, separate from the primary tumor, and attack the surrounding tissues and distant organs through the blood and lymphatic circulation systems (20). Many neoplastic illnesses are associated with uncommon angiogenesis, which has a significant impact on the growth and metastasis of tumors (21). To date, a variety of therapy approaches have been used to inhibit metastasis. Quercetin can decrease the cancer metastasis by suppressing various molecular pathways and angiogenesis (22).

Tumor angiogenesis and metastasis are caused by the interaction of VEGF and VEGF receptor 2 (VEGFR2), as well as by signals that are released in response to this process (23, 24).

Epithelial to mesenchymal transition is another factor in the development of metastasis (EMT). This process is associated with increased expression of mesenchymal markers including N cadherin, vimentin, and snail and decreased expression of epithelial proteins such as E cadherin and MUC1 (25). According to a previous study, quercetin inhibited tumor growth in prostate cancer by targeting the angiogenic pathway which mediated by VEGFR-2 and as a result inhibiting the expression of the AKT/mTOR/ribosomal protein S6 kinase (P70S6K) regulatory factor (17). Another investigation reported the effects of quercetin on inhibition of EMT, angiogenesis, and invasiveness through the epidermal growth factor receptor (EGFR)/VEGFR-2-mediated pathway in breast cancer (26). Matrix metalloproteinases (MMPs) are mentioned as compounds that play a special role in cancer invasion and metastasis processes by destroying the components of extracellular matrix (ECM) (27) (19). MMP2 and MMP9 are two remarkable compounds in metastatic breast cancer (28–30). In addition, a study investigated the effect of quercetin on breast cancer cell lines (MDA-MB-231) and showed that after treatment with this flavonoid, the expression of these two proteinases decreased (19). In studies related to colorectal cancer and squamous cell carcinoma of the head and neck (HNSCC), the inhibitory effect of quercetin on the migration of tumor cells has been shown by regulating the expression of MMPs (31, 32). According to the results obtained from the laboratory studies, quercetin by inhibiting the Akt activation pathway dependent on Snail, diminishing the expression of N-cadherin, vimentin, and ADAM9 and raising the expression of E-cadherin and proteins related to MMPs significantly inhibited the invasion and metastasis of lung cancer cells (33). In an examination of pancreatic cancer, quercetin inhibited the EMT, reduced invasive and metastasis, and reversed IL-6-induced EMT and MMP expression by inhibiting STAT3 signaling (34). Besides, quercetin 3-O-glucoside inhibited EGFR signaling, resulting in the suppression of EGF-induced migration in pancreatic cancer cells (35).

Transforming growth factor-β (TGF-β), as an inducer of EMT plays a vital role in prostate cancer metastasis. According to the research of Meghna M. Baruah, quercetin prevented EMT caused by TGF-β by reducing the expression of TGF-β caused by vimentin and N-cadherin, Twist, Snail, and Slug and increasing the expression of E-cadherin in PC-3 cells. The result of this process was the control of prostate cancer metastasis by this flavonoid (36).

In metastasis and progression of melanoma, activation of c-Met receptor tyrosine kinase has an important role. Hepatocyte growth factor (HGF)-mediated activation of C-Met signaling has been proposed as a therapeutic aim for melanoma metastasis. A group of researchers stated that quercetin showed anti-metastatic activity in melanoma by inhibiting c-Met phosphorylation, reducing its homodimerization, and ultimately suppressing the HGF/c-Met signaling pathway (37).

## Anti-proliferation effect of quercetin on cancer

p53 protein, as a tumor suppressor, plays an important role in preventing cancer by regulating the cell cycle, apoptosis, and DNA repair (38). In this regard, has been stated that quercetin induced cell cycle arrest and apoptosis in hepatocellular carcinoma cells (HCC) by stabilizing or inducing p53 (39). Another experiment exhibited that quercetin exerted an anti-proliferative role on HCC cells by lessening intracellular ROS independently of p53 expression (40).

Protein kinase C (PKC) and phosphatidyl 3-kinase (PI3K) also play an influential role in increasing cell proliferation and survival. In Akhilendra Kumar Maurya’s study, quercetin prevented the proliferation of cancer cells by increasing the expression of p53 and BAX in hepatocellular carcinoma (HepG2) cell lines through the reduction of PKC, PI3K, and cyclooxygenase (COX-2) (41). During a study on melanoma cells, A375, it has been found that quercetin can inhibit cell proliferation by regulating proteins of the Wnt/β-catenin signaling pathway, DVL2, β-catenin, cyclin D1, Cox2, and Axin2 (42). By examining the effect of quercetin on the cell cycle and growth of human gastric cancer cells, it was determined that this compound has led to the multiplicity of this type of cancer by inhibiting the cell cycle in the G1 phase (43).

## Antiapoptotic effects of quercetin on different types of blood cancers

### *In vitro* studies

Leukemia is one of the most common malignant diseases affecting the world’s population (7). ALL is the most commonly diagnosed cancer in the childhood age group and accounts for approximately 25-30% of all malignant childhood diseases. The incidence of ALL in the first year of life is slightly higher in females than in males (44). Although the exact cause of ALL is unknown, leukemia cells have several genetic and chromosomal abnormalities and epigenetic changes that can disrupt the cell’s signaling pathways and transcription factors (45–48).

AML, as an aggressive form of leukemia, generally targets regulated processes of differentiation and growth of the human hematopoietic myeloid lineage (6). The incidence of AML has two peaks, early childhood and adulthood. The average age of newly diagnosed AML patients is 66 years. The disease can appear at any age, but diagnosis before age 40 is relatively rare (49).

CLL is a disease of neoplastic B cells, whereas the entity formerly described as T-CLL is now called T-cell prolymphocytic leukemia (50). It is a disease of the elderly with a median age of 72 years at diagnosis and 79 years at death from CLL (51).

CML progresses from the chronic phase, which characterizes the Philadelphia chromosome as the only genetic abnormality, to blast crisis, often accompanied by secondary changes in chromosomes and molecules (52).

The rate of malignant lymphoma worldwide is estimated to be 3-4%. Lymphoma can be divided into two major groups: Hodgkin lymphoma (20-30% of total lymphoma) and non-Hodgkin lymphoma (HL or NHL). Historically, attempts to understand the etiology of NHL have been hampered by its heterogeneous composition with different clinical behaviors and numerous subtypes with histopathological and immunological phenotypes. Over time, attempts have been made to integrate this non-uniformity into the classification system (53).

One study investigated the effects of quercetin on the induction of apoptosis in CCRF-CEM human T-cell acute lymphoblastic leukemia (T-ALL), HL-60 human acute promyelocytic leukemia (APL), and K-562 human chronic myeloid leukemia cells (CML). A dose-dependent reduction in survival was observed when quercetin was administered to leukemia cells, along with an increase in apoptosis and necrotic cell death. Increasing quercetin levels reduced the proportion of viable cells in all three leukemia cell lines. Still, the duration of treatment with quercetin did not appear to make a significant contribution to apoptosis and necrosis. Since K562 cells do not undergo apoptosis or necrosis, quercetin is more likely to suppress the time-dependent suppression of CCRFCEM and HL60 cells than K562 cells when constant quercetin concentrations are used over time. In each of the three leukemia cell lines, the proportion of necrotic cells was lower than the proportion of apoptotic cells. Quercetin-induced apoptosis in leukemia cells reached up to 22%, while the proportion of apoptotic cells with the maximum was about 60%. A decrease in survival rate was observed in leukemia cell lines after treatment with quercetin (54).

In another study, MOLT4 (a T cell line that causes ALL) and a Raji cell line (derived from human lymphocytes) were used as blood cancer models to study the effect of quercetin on leukemia. The MTT assay showed that the inhibitory effect of each concentration of quercetin on the survival of all cancer cell lines increased with increasing incubation time. Subsequently, apoptosis rates were assessed in a panel of 2 cell lines with high and low sensitivity to quercetin (MOLT-4 and Raji, respectively). The results showed that quercetin initiates the process of apoptosis in these cells in a dose-dependent manner (55).

Kawahara examined quercetin’s apoptotic effect on AML models HL60 and TMD7, acute promyelocytic leukemia (NB4), erythroleukemia (HEL), chronic myeloid leukemia (K562), Jurkat, KOPT-K1, DND-41 (as T-ALL models), Burkitt lymphoma (DND-41), and DLBCL TMD8. The quercetin inhibitor suppressed apoptotic cells and nuclear condensation in cultures that suppressed growth, which demonstrated quercetin suppresses the development of many leukemia and lymphoma cells (56).

**Figure 1:**
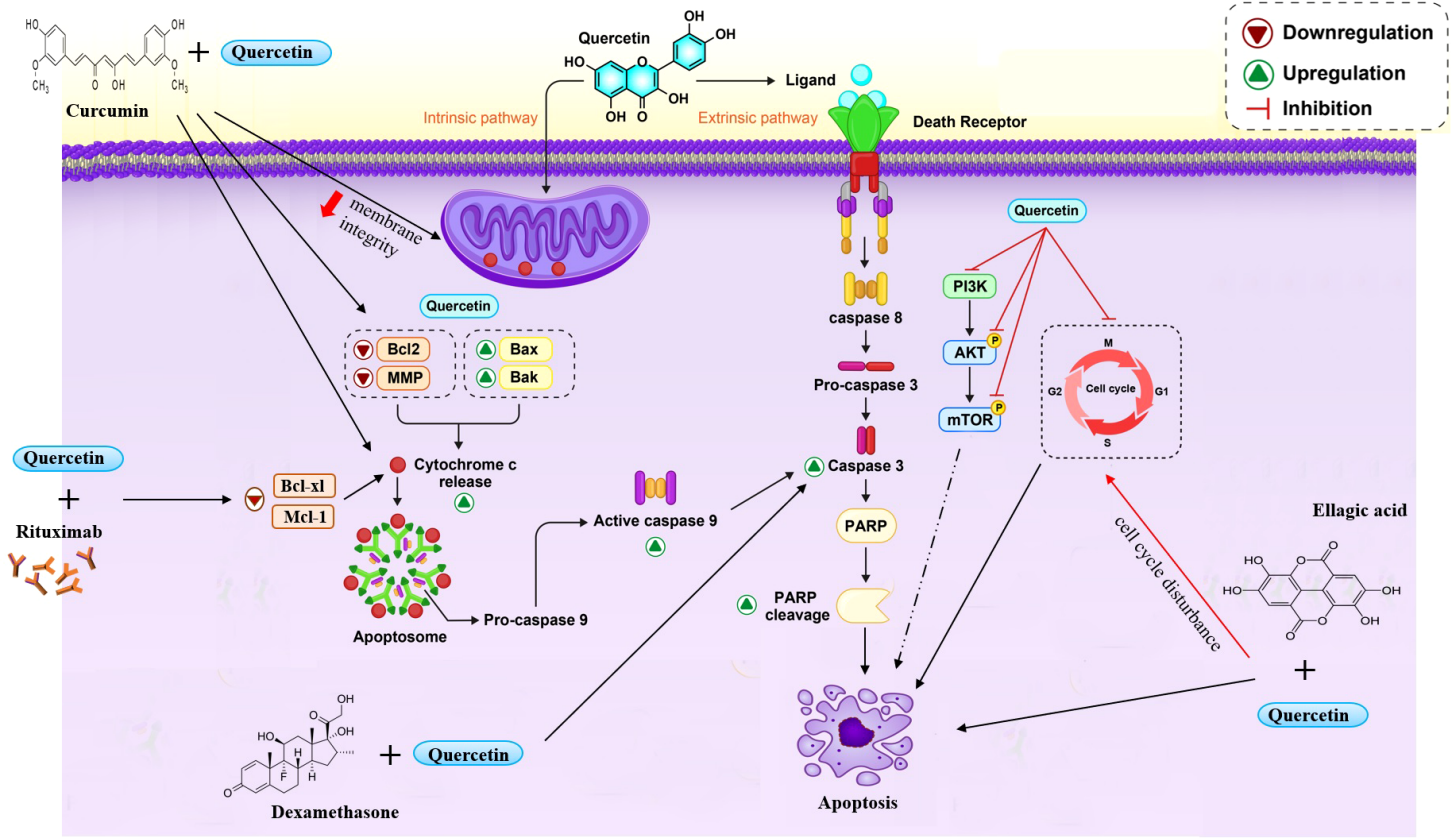
Quercetin induce cell apoptosis through different ways. Quercetin induced c-cytochrome-dependent apoptosis and caspase-3. Also quercetin suppressed cell cycle in different phases and inhibited the phosphorylation of PI3K/Akt and mTOR. Co-treatment quercetin with some compounds improved the efficacy and increased apoptosis.

In 2012, to study the effect and mechanism of quercetin on the apoptosis of leukemia cells, the HL60 leukemia cell line was treated with various doses of quercetin (57). Quercetin induced apoptosis in leukemia cells by decreasing the expression of PI3K and Bcl2 proteins, inhibiting Akt phosphorylation, enhancing BAX protein levels, increasing caspase 2 and 3 activation, and increasing cleavage of poly (ADP ribose) polymerase. Their results showed that apoptosis caused by quercetin was mediated by decreased pAkt and Bcl2 levels, elevated BAX levels, and activation of the caspase family in HL60 cells (57) (**Figure 1**).

A research study has shown that quercetin combined with a recombinant ligand affecting apoptosis, such as TNF-related apoptosis-inducing ligand (TRAIL), or anti-CD95, could sensitize several leukemia cell lines and B cells isolated from patients with B-CLL. The expression and activity of antiapoptotic proteins belonging to the Bcl-2 family were shown to confer an apoptotic resistance on myeloid cell leukemia-1 (Mcl-1). Quercetin in cell lines and B-CLL correlates with the expression and activity of the Mcl-1 antiapoptotic protein belonging to the Bcl-2 family. Spagnuolo’s investigation exhibited that quercetin negatively regulates mRNA and protein levels in the U-937 cell line (human myelomonocytic), and Mcl-1 affects mRNA stability and proteolysis. Their trial results advocated the possibility of using quercetin as adjuvant chemotherapy in CLL or other cancers (58, 59).

To determine the antitumor effects of quercetin in AML, several investigators used MV411 and HL60 as two myeloid leukemia cell lines exposed to different quercetin levels. Quercetin-induced apoptosis is dependent on a decrease in the mitochondrial membrane potential (MMP) and the Bcl2 protein. The results displayed that quercetin cleaved caspase 8, caspase 9, caspase 3, and PARP dose-dependent on MV411 cells. Following, to define the assistance of these caspases to quercetin -induced apoptosis in MV411 cells, ZIETDFMK (a caspase8 inhibitor), ZLEHDFMK, and ZVADFMK were used to assess their protecting effect on apoptosis with quercetin. Finally, Huan et al.’s research revealed that quercetin inhibited the proliferation of MV411 and HL60 cells. Hoechst’s staining results demonstrated that quercetin induced cell death by apoptosis in AML cells (59).

Primary effusion lymphoma (PEL) is an aggressive B-cell lymphoma whose pathogenesis is closely related to Kaposi’s sarcoma-associated herpesvirus (KSHV) (60, 61). In Marisa Granato’s study, all three PEL cell lines (BC3, BCBL1, and BC1) showed a dose-dependent decrease in cell survival and growth after treatment with quercetin (62). They found that quercetin by inhibiting PI3K/AKT/mTOR and STAT3 pathways, decreasing the expression of cellular proteins such as c-FLIP, cyclin D1, and c-Myc, as well as reducing the production of IL-6 and IL-10 cytokines, leads to the death of PEL cells (62).

Examination revealed that quercetin caused marked inhibition of K562 cell growth and mild cytotoxicity (63). In addition, quercetin induced c-cytochrome-dependent apoptosis and caspase-3 almost exclusively in the HSB2 cell line. Exposure of K562 cells to quercetin also significantly raised the cells in the G2/M phase, which reached a maximum peak in 24 hours (64).

Using HL60 and U-937 cell lines *in vitro*, Marisa Claudia Alvarez et al. investigated the molecular mechanisms underlying quercetin’s apoptotic effect by evaluating the impact of quercetin treatment on DNA methylation and post-translational histone modifications of genes involved in the apoptosis pathway (65). According to them, quercetin-induced cell death is associated with increased transcription in the promoters of genes involved in the apoptotic pathway through DNA demethylation activity, histone deacetylase (HDAC) repression, and H3ac and H4ac enrichment (53).

In Nalm6 as a leukemia cell line, the cytotoxic effect of quercetin was examined. The results indicated a specific S phase cessation after 16 h of quercetin treatment in these cells (66). Furthermore, a dose-dependent boost in the apoptotic cell population was observed in the Sub-G1 phase when Nalm6 cells were treated with rising concentrations of quercetin. Interestingly, S-phase cell cycle arrest was observed only at a lower time point (16 hours), while a dose-dependent increase in the sub-G1 population was observed after 2 days. Therefore, the findings suggest that quercetin induces S-phase arrest following apoptosis of cancer cells. Their data also suggest quercetin-mediated cytotoxicity involves the mitochondrial pathway of apoptosis. DNA damage and apoptosis in cells are caused by ROS assembly after antitumor treatment. Their results revealed that quercetin did not induce notable modifications in ROS levels, so quercetin-induced cell death was not due to ROS production. The mechanism by which quercetin induces apoptosis was due to increased p53 and p-p53 (phosphor-p53) associated with cleavage of the apoptosis marker MCL-1. Also, lessened levels of the anti-apoptotic proteins BCL2 and BCL-XL and heightened levels of the apoptotic protein BAX were observed. In addition, the departure of cytochrome C in association with SMAC/DIABLO exhibited activation of mitochondrial endogenous signaling pathways conducting apoptosis. These findings were further supported by enhanced levels of activated caspase 3, cleaved caspase 9, and PARP1 (64).

In addition, another study evaluated the apoptosis impact of quercetin on 232B4 CLL cells. The results exhibited that quercetin decreased 232B4 proliferation dose-dependently compared with untreated control samples. The cause of the apoptosis effect of quercetin on 232B4 cells was modifications in caspase3 enzyme activity and loss of MMP. Thus, quercetin induces apoptosis in 232B4 cells (67).

To assess quercetin’s effect on the rhTRAIL–induced apoptosis of human myeloid leukemia KG‐1 cells. The XIAP, cIAP1, and cIAP2 genes were evaluated to determine mRNA expression levels in the treated and untreated groups. Based on the results presented, it was concluded that XIAP mRNA expression levels did not differ significantly in any group at 12 hours after treatment. In addition, no changes were observed in XIAP gene expression in the rhTRAIL-treated group, 1 and 2 days after treatment compared to the control group. They found that quercetin treatment of KG1 cells could significantly downregulate XIAP mRNA expression levels at 24 and 48 hours, either with quercetin alone or with rhTRAIL treated cells.

Their results showed that quercetin sensitized KG-1 cells to TRAIL-induced apoptosis. This is accomplished by increasing the expression level of the death receptor gene and decreasing the expression of anti-apoptotic proteins (68).

### *In vivo* studies

Quercetin has been reported to affect different aspects of the biological function of the cell, like cell cycle arrest, autophagy, angiogenesis, metastasis, and apoptosis. The section of this article is a review of all evidence available from *in vivo* studies in which quercetin affects non-solid cancer.

It is worth mentioning that quercetin can block the survival of radio-resistant B-1 cells in the peritoneal cavity of immune-deficient mice. It was also detected that using quercetin restored the levels of miR-15a and miR-16 and decreased BCL-2 expression, and these factors can abolish the ability to expand or migrate to other organs in eradicated B-1 cells *in vivo*. This result suggests that quercetin could be an essential factor in the restitution of the apoptosis sensibility of B-1 cells (69).

Alvarez et al. found that DNMT protein levels can eliminate quercetin due to the downregulation of STAT3 and p-STAT3. A subcutaneous engraft with HL60 and/or U937 cells (31) decreased the expression of class I HDAC1 and HDAC2, but the mRNA expressions of DAPK1, BCL2L11, BAX, BNIP3L, and APAF1 increased sharply compared to xenograft models and mice (70).

A mixture of P93 cell suspension and quercetin was applied after 21 days to female NOD mice with the KDCSC/J lineage. In this study, activation of apoptotic markers decreased BCL2, BCL-xL, and McL-1 expression by affecting BAX expression. Also, Beclin-1, PI3K, Atg5-Atg12, and Atg7, the protein expression related to autophagy increased, and in cell-cycle, phosphorylated Rb, cyclin D, cyclin E proteins expression decreased, and p21 has a different destiny compared with the control group after quercetin treatment (31). Another study observed that tumor burden was lower with exposure to quercetin in combination with ARP-1 cells compared with the control group. Also, injection of that flavonoid combined with dexamethasone had a similar effect to the group treated with quercetin alone. Significantly, quercetin can be interfered with by activating caspase-3 and p21, inhibiting the proliferation of multiple myeloma cells and reducing c-Myc expression in the human NOD-SCID-MM mouse model (71).

In a partly similar study, the administration of quercetin in U937 xenografts was investigated. Their results showed that McL-1 and BAX could stimulate apoptosis in human leukemia by McL-1 down-regulation, BAX conformational change, and mitochondrial translocation, which resulted in cytochrome *C* release. On the other hand, reversion of quercetin-induced apoptosis and abrogation of active caspase and apoptosis were induced by knockdown of BAX and did not affect quercetin-induced apoptosis by McL-1 downregulation, but interruption of McL-1 augmented BAX activation and lethality motivated by quercetin (72).

In the human leukemia study*, Lee et al.* In NOD-SCID mice with HL-60 tumor xenografts, quercetin significantly reduced tumor cell growth *via* the ERK pathway (73).

In a similar article, utilization of green tea and quercetin as monotherapy caused the reduction of levels of anti-apoptotic proteins, CDK6, CDK2, CYCLIN D/E/A, BCL-2, BCL-XL, and MCL-1 and an increase in expression of BAX. Furthermore, injections of dual quercetin and green tea into HL-60 xenografted mice activate caspases, promoting the G1 phase of the cell cycle (**Figure 1**).

Also, it has been shown that both green tea and quercetin upregulated the expression of all BECLIN1, ATG12-ATG5 autophagy-related proteins. To further characterize this process, the conversion of LC3-I to LC3-II was assessed as a hallmark of autophagy. Moreover, quercetin inhibited tumor growth in mice grafted with HL60 human leukemia cells.

Other animal studies showed quercetin administration before and during treatment with etoposide could reduce bone marrow hypoplasia and the level of oxidative DNA damage in rats. The number of myeloid precursors and nucleated erythroid cells was significantly increased by pre-treatment with quercetin followed by etoposide compared to appropriate controls.

The others demonstrated the effects of WEHI-3 cells treated with Quercetin in BALB/c mice. In this case, the percentage of Mac-3, CD11b markers, and the weight of the spleen and liver decreased, but CD19 levels, phagocytic cells like macrophages isolated from the peritoneum, and natural killer cells increased, stimulated, and promoted respectively (74).

**Figure 2:**
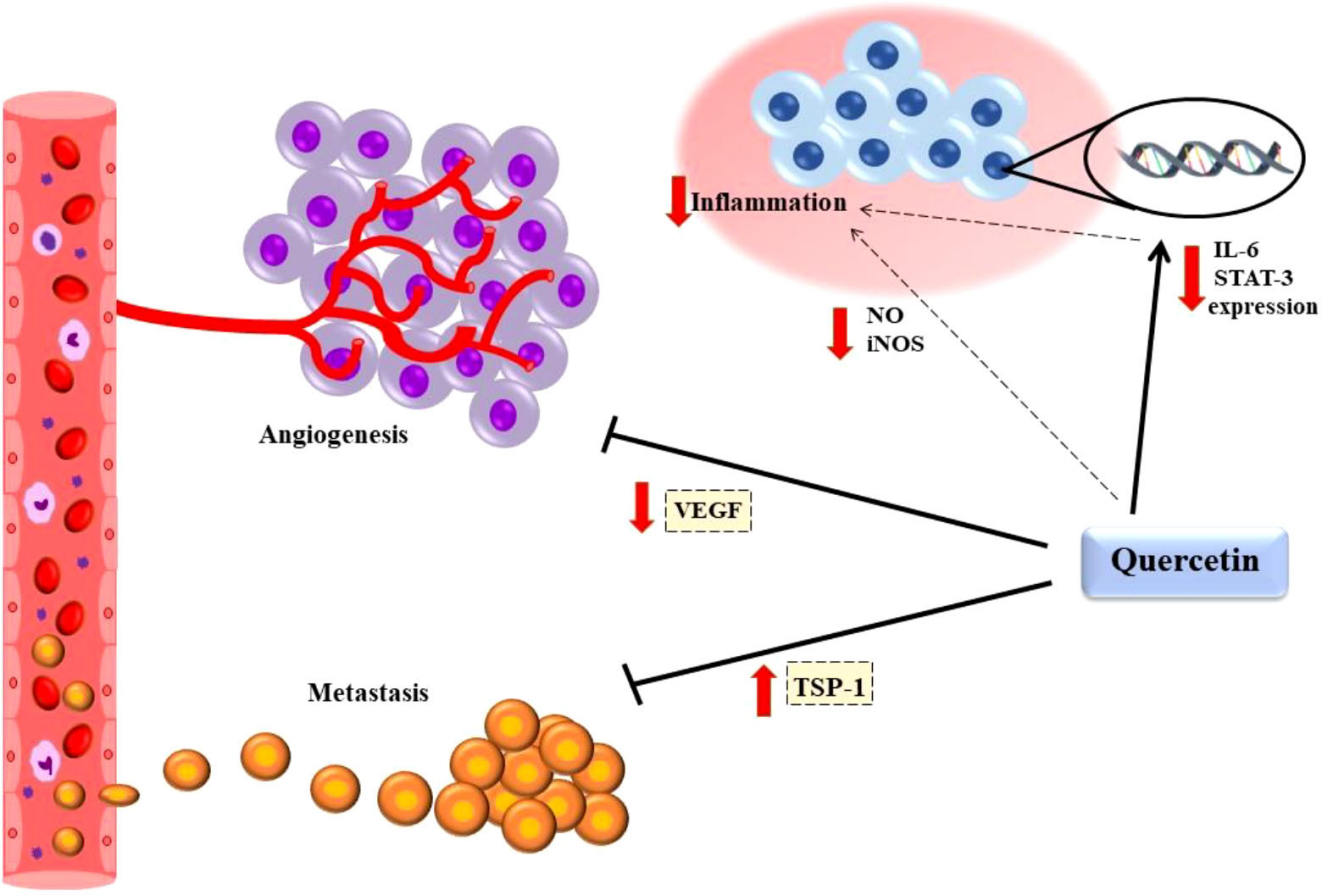
Quercetin had anti-tumor and anti-inflammatory effects. Quercetin declined inflammatory condition through decreasing VGEF-A, NO, iNOS. Also, It increased TSP-1 mRNA and protein expression to prevent angiogenesis and suppressed metastasis by reducing VEGF secretion. Vascular endothelial growth factor A (VEGF-A); Nitric oxide (NO); Inducible nitric oxide synthase (iNOS); Thrombospondin-1 (TSP-1).

Quercetin has multi-effects on a dose-dependent mouse model of T cell lymphoma (Dalton’s lymphoma). It could decrease the phosphorylation of AKT, PDK1, and downstream factors such as pro-apoptotic proteins BAD, mTOR, IκBα (NF-κB regulator), and GSK-3β that have a role in cell survival. Quercetin upregulates the level of PTEN as a tumor suppressor, which inhibits AKT signaling. Moreover, Quercetin had anti-inflammatory and anti-angiogenesis effects, decreasing VGEF-A, NO, iNOS, and COX-2 levels (75) (**Figure 2**).

## Effects of co-treatment quercetin with other compounds

In many studies, the properties of quercetin, such as anti-inflammatory, anti-carcinogenic, and growth-suppressing effects, were analyzed in various cell lines of different cancers (76). Studies on the impact of combining quercetin with other compounds and drugs can be considered. He et al. Found that combining quercetin with dexamethasone promoted apoptosis in multiple myeloma cell lines (RPMI8226, ARP-1, and MM.1R). So, they had a synergistic effect on myeloma treatment. Also, in xenograft models, this combination enhances caspase-3 activity. However, the reduction of tumor burden didn’t differ in the quercetin combined with dexamethasone group compared to quercetin individually (71).

In combination with chloroquine (lysosome suppression), quercetin increases cell apoptosis (77).

The use of quercetin and chemotherapy can potentiate their effect on the malignant cell. Russo et al. Shown that CLL isolated-B cells treated with quercetin and anti CD95 increased cell death compared to a single treatment with quercetin or Anti CD-95. Furthermore, they reported quercetin improves the efficacy of Fludarabine which is used in CLL treatment, and suppresses cell growth through DNA synthesis disruption. Quercetin and Fludarabine can enhance apoptosis compared to monotherapy and reduce B-CLL cell resistance to Fludarabine (78, 79). In other studies, Li et al. Reports suggest quercetin could boost the impact of rituximab (anti-CD20 antibody) on diffuse large cell lymphoma (DLBCL) cell lines. The results showed that quercetin combination with rituximab increased apoptosis and cell growth inhibition compared with rituximab mono treatment in WSU-DLCL2 and Sudhl4 cell lines. Moreover, quercetin could potentiate the anti-tumor effect of rituximab through the STAT3 pathway. Treatment with combination agents further suppresses phosphorylated STAT3 and decreases the expression of genes associated with survival, such as Mcl-1 and Bcl-xl (80).

Vincristine (VCR) is an anti-neoplastic agent in NHL treatment. Zhu et al. Investigated the effect of Quercetin and VCR that were loaded on lipid-polymeric nanocarriers (LPNs). Que/VCR LPNs had a synergistic effect and decreased cell viability in Raji cells (a human Burkitt’s lymphoma cell line). So, quercetin increased the cytotoxicity of VCR. Also, they injected Raji cells into BALB/c mice to develop a tumor and then used Que/VCR LPN and Que/VCR solution. They found that tumor growth and tumor size decreased after treatment with two forms, Que/VCR, compared to a single therapy, and Que/VCR LPN had better efficacy than Que/VCR solution (81).

In combination with hyperthermia, Shen et al. Quercetin is a method used in cancer treatment by heating, and it was found to reduce Doxorubicin hydrochloride resistance in leukemia cell line K562 (82). So, quercetin may sensitize malignant cells to chemotherapy agents and potentiate their impacts. This capability needs to be further investigated.

For polyphenols, a class of natural compounds, treatment with ellagic acid, luteolin, and curcumin alone showed excellent anticancer effects. However, combined with quercetin, some adverse effects were observed, such as ROS increase, apoptosis promotion, and proliferation reduction. Mertens-Talcott et al. Observed that quercetin interaction with ellagic acid-induced synergistic effect led to cell cycle disturbance, apoptosis promotion, and proliferation reduction in the MOLT-4 human leukemia cell line (83–85).

Luteolin is another polyphenol flavonoid with anti-inflammatory and anti-cancer properties. In CLL cell lines HG-3 and EHEB, the cytotoxic impact of luteolin is enhanced in the presence of quercetin. The IC50 value of luteolin was decreased with increasing concentrations of quercetin (10, 20 and 30 M).

Curcumin is a natural anti-inflammatory and anti-tumor compound that is present in the spice turmeric (86). Studies showed that curcumin could suppress proliferation in CML cells. Altundag and colleagues found that the combination of quercetin and curcumin enhanced reactive oxygen species (ROS) generation compared to curcumin treatment alone in the CML cell line. Also, they found that co-treatment with quercetin and curcumin led to a reduction of mitochondrial membrane integrity, promotion of cytochrome C release, and apoptosis induction in CML cells (87). The combination of these two compounds has a similar effect on other cancer cell lines. Srivastava et al. results showed that in a melanoma cell line (A375), quercetin combined with curcumin displayed a higher suppressor effect on cell proliferation through Wnt/β-catenin signaling and decline of BCL-2 and caspase 3/7 induction. In addition, these compounds exhibit synergistic anti-proliferative effects on colon, lung, and breast cancer cells (HCT116, A549).

Notch, Hedgehog (Hh), and Wnt signaling pathways have the potential capacity for the growth of malignant hematopoietic stem cell clones. Cell growth can be suppressed by inhibitors of these pathways. In combination with quercetin, a Wnt inhibitor, and *-secretase inhibitors (GSIs), these drugs can suppress growth synergistically and increase the decline of Notch1 in the DND-41 T-ALL cell line. Also, in combination with the Hh inhibitor cyclopamine, quercetin has an additive effect on suppressing leukemia cell growth (88).

Quercetin promoted the activity of bortezomib (proteasome inhibitor) in the Primary Effusion Lymphoma PEL cell line, which is an aggressive B cell lymphoma. Cotreatment of quercetin and bortezomib led to the decline of cell survival compared to monotherapy in BC3 and BCBL1 cell lines (62).

Many *in vitro* studies indicate that quercetin is anti-carcinogenic, which suggests that when combined with other drugs, quercetin might be an effective treatment for cancer patients. A summary of previous studies on the therapeutic effects of quercetin on various types of blood cancer is listed in **Table 1**.

**Table 1:** The therapeutic effects of quercetin on blood cancer.

Model	Type of Quercetin (Que)	Cell/Animal model	Result	Que Dose	Ref
*In vitro*	Que	CCRF-CEM, HL-60, K-562	resulted in a dose-dependent decrease in survival and increased apoptosis and necrotic cell death in of leukemia cells.	12·5, 25, 50, 75 μM	(54)
*In vitro*	Que	MOLT4, Raji	induced apoptosis rates in both cell lines at the utilized concentrations.	10, 20, 40, 80, 120 µM	(55)
*In vitro*	Que + cyclopamine	HL60, TMD7, NB4, DND-41, Daudi, TMD8	suppressed cell growth and induced apoptosis in all cell lines by its effects on Wnt and Notch signaling.	25, 50 μM	(56)
*In vitro*	Que	HL60	induced apoptotic processes by the decrease of pAkt and Bcl-2 levels, the increase of BAX level, and the activation of caspase families.	0, 12.5, 25, 50, 100, 150, 200 μM	(57)
*In vitro*	Que	PEL cells (BC3, BCBL1, BC1)	induced apoptosis and autophagy in primary effusion lymphoma cells by inhibiting PI3K/AKT/mTOR and STAT3 signaling pathways.	0, 12.5, 25, 50, 100 μM	(62)
*In vitro*	Que	K562, HSB2	raised the cells in the G2/M phase and inhibition of cell growth and mild cytotoxicity of in the K562 cell line. induced c-cytochrome-dependent apoptosis and caspase-3 in the HSB2 cell line.	0, 25 μM	(64)
*In vitro*	Que + Resveratrol	232B4	caused dose dependent inhibition of cell proliferation and increased apoptotic cell population through induction of caspase-3 activity	0.1, 1, 10, 50 μM	(67)
*In vitro*	Que	KG‐1	increased the apoptotic effects of TRAIL against the acute myeloid KG‐1 cells	10, 50, 100,150, 200, 250, 300 μM	(68)
*In vitro*	Que	HL60, U-937	increased transcription in the promoter regions of genes involved in the apoptotic pathway through DNA demethylation activity, histone deacetylase (HDAC) repression, and H3ac and H4ac enrichment	50 μM	(70)
*In vitro*	Que	CEM, K562, Nalm6	induced S-phase arrest following apoptosis of cancer cells and involvement of the mitochondrial pathway of apoptosis during quercetin -mediated cytotoxicity	10, 50, 100, 250 μM	(66)
*In vitro*	Que	U-937	proapoptotic activity of quercetin by downregulating Mcl-1 acting directly or indirectly on its mRNA stability and protein degradation,	0.5–5 μM	(58)
*In vitro*	Que	HL-60, MV4-11	induced cell death *via* downregulation of VEGF/Akt signaling pathways and mitochondria-mediated apoptosis in AML cells.	0, 12.5, 25, 50, 100 μM	(89)
*In vivo*	Que	NOD/SCID mice	Que could be an essential factor in the restitution of apoptosis sensibility of B-1 cells	100 μM	(69)
*In vitro*	Que + Fludarabine	HL-60, MV4-11	Quercetin significantly enhanced anti-CD95- and rTRAIL-induced cell death as shown by decreased cell viability, increased caspase-3 and -9 activities	10-25 μM	(78)
*In vitro*	Que + Rituximab	DLBCL, WSU-DLCL2, Sudhl4	synergistically enhanced rituximab-induced growth inhibition and apoptosis. Que applied inhibitory activity against STAT3 pathway and downregulated the expression of survival genes.	10, 20, 40, 80 μM	(80)
*In vitro+ In vivo*	Que + Dexamethasone	RPMI8226, ARP-1, MM.1R	inhibited proliferation by inducing cell cycle arrest in the G2/M phase and promoted apoptosis. inhibited tumor growth in xenograft model.	–	(71)
*In vitro+ In vivo*	Que	U937, Jurkat, HL-60	induced apoptosis in leukemia versus normal hematopoietic cells, through a process involving Mcl-1 down-regulation, which in turn potentiates BAX activation and mitochondrial translocation, culminating in apoptosis.	40 μM	(72)
*In vitro+ In vivo*	Que	HL-60	induced cell death of HL-60 cells *in vitro* and *in vivo* through induction of intracellular oxidative stress following activation of an ERK-mediated apoptosis pathway	100 μM	(73)
*In vivo*	Que + Green tea	HL-60 xenografts	induced signaling at the level of apoptosis, cell cycle and autophagy which converge to antigrowth effects in HL-60 xenograft mice	120 mg/kg	(90)
*In vivo*	Que	WEHI-3	decreased the percentage of Mac-3 and CD11b markers, suggesting that the differentiation of the precursors of macrophages and T cells was inhibited.	2,4 mg/kg	(74)
*In vitro+ In vivo*	Que + Vincristine (VCR)	Raji	The formulated VCR/Que LPNs exhibited nano-size, negative zeta potential with sustained release profile *in vitro*. The dual drug loaded LPNs exhibited the best antitumor efficacy *in vitro* and *in vivo*.	50 mg	(81)

According to the anti-carcinogenic feature of quercetin in many *in vitro* studies, it seems quercetin combined with other drugs will be a potential choice in clinical trial studies in cancer patients. Summary of previous studies on the therapeutic effects of quercetin in various types of blood cancer listed in **Table 1**.

## Lung cancer

A high global incidence rate and cancer-related deaths are associated with lung cancer, with 1.76 million deaths worldwide (91). In the United States, lung cancer has been reported as the most commonly diagnosed cancer, with an incidence rate of 71.3 and 52.3 per 100000 in men and women, respectively, in 2019 (92). The morphological and genetic features of non-small cell lung cancer (NSCLC) and small cell lung cancer (SCLC) in lung carcinoma have been correlated (93). Flavonoids have shown ineffective treatment for various cancers, especially lung cancer, in some studies. Among phytochemicals, quercetin displays a vital role in cancer-related molecules such as apoptotic proteins, cycle-dependent kinases (CDKs), cyclins, matrix metalloproteinase (MMPs), and growth factors *via* signaling pathways (94). The purpose of this article is to summarize a number of studies that focus on the role of quercetin in human lung cancer treatment, diagnosis, and prognosis.

## Potential anti-cancer effects of quercetin in lung cancer

### *In vitro* studies

Multiple *in vitro* studies have noticed quercetin can improve and regulate many signaling pathways during cancer development. Also, it plays a key role in apoptosis, survival, angiogenesis, inflammation, and cell cycle. Therefore, studies showed that quercetin could suppress or downregulate molecules in various signaling pathways *via* the effect on apoptosis (94). It was found that treatment with quercetin at 10, 30, and 60 mM was investigated in a human A-549 cell line. Quercetin can affect microfilaments, microtubules, and vimentin filaments along with inhibition of vimentin and N-cadherin (cytoskeletal proteins) expression that function in migration and induce an obvious increase in BCL2/BAX-mediated apoptosis (95). A previous study reported that quercetin could downregulate IL-6, STAT-3, Bcl2 activity, NF-kB expression, and upregulate the Annexin and the PI cell population through induction of mitochondrial-mediated apoptosis in A549 cells in non-small-cell lung cancer (NSCLC) (96) (**Figure 2**). Additionally, it was suggested that Tumor necrosis factor (TNF)-related apoptosis-inducing ligand (TRAIL) -induced cell death was elevated by quercetin *via* activation of autophagy flux and apoptosis. Regarding the above subjects, quercetin can reduce the P62 level, interrupt Akt signaling, and enhance caspase-3 cleavage in A-549 cells (97). An integral part of the tissue is the extracellular matrix (ECM), which plays an important function in lung cancer (94). In a detailed study using quercetin, maximum inhibition of invasion was reported by quercetin-metabolite-enriched plasma (QP) in A-549 cells related to the amount of dose (47%, p<0.05). Like invasion assay, QP significantly inhibited migration, Matrix metalloproteinases (MMP-2) activity, and MMP-2 protein expression in dose-dependent effects (52%, 59%, and 62%, respectively). furthermore, using 10 mM quercetin-3-glucuronide (Q3G) and quercetin-3-sulfate (Q3S) caused a high expression of nm23-H1(84% and 42%, respectively), as well as elevated and tissue inhibitor of metalloproteinase (TIMP-2) (31% and 13% respectively). In addition, these metabolic of quercetin markedly increased peroxisome proliferator-activated receptor gamma (PPAR-g), a member of the nuclear hormone receptor, expression (71% and 54% respectively), while quercetin had no effect (98). A-549 cells were shown to have reduced mRNA expressions of urokinase plasminogen activator (uPA), Upar, protein expression of CXCR-4, CXCL-12, SDF-1 when quercetin was applied at 20 and 40 mM/ml by real-time PCR. The mRNA expression of CXCL-8, CXCR1 was inhibited in a study comparing lung cancer cells with a control group in terms of migration and invasion during epithelial to mesenchymal transition. Moreover, in both A-549 and HCC827 cell lines after quercetin treatment for 24h, wound closure, migratory, and invasive abilities were suppressed at doses of 10-50mM. furthermore, quercetin can suppress Snail, which is crucial for activation of EMT and cell motility. Also, upregulation of Snail and p-Akt resulted in promoting lung cancer progression. On the other hand, overexpression of Snail can decrease spine expression and elevate Akt activation (33). One of the flavonoids that can suppress growth tumor is quercetin. lung cancer growth can suppress by quercetin *via* binding aurora B. Aurora B is defined as a serine-threonine kinase and a protein that plays a role in the attachment of the mitotic spindle to the centromere. Moreover, it has a noticeable expression in cancerous cells related to cancer development and progression.

Zhu Xingyu et al. reported that quercetin could bind to aurora B directly, which leads to inhibition of its activity. Also, using different concentrations of quercetin with high, middle, and low expression of aurora B in A-549, H1975, and H441, respectively, indicated unequal results, which A-549 cells with high aurora B expression were more sensitive to quercetin. further, epidermal growth factor (EGF) is suppressed by different concentrations of this flavonoid, which causes inhibition of several colonies (more than 30%) in JB6 Cl41 cell (99). Further, the human NSCLC cell line A-549 encountered Hyperoside (quercetin-3-O-β-D-galactopyranoside) in 0.5, 1, and 2 mmol/L doses. This assay showed that the phosphorylation of Akt, mTOR, P70S6K, and 4E-BP1 were inhibited, which contributes to the anticancer of hyperoside in autophagy, but that the phosphorylation of ERK1/2 and expression of LC3-II (anti-microtubule-associated protein 1 light chain 3 (LC3) were increased in A-549 cell (100). Some results about Claudin-2, which is expressed markedly in lung adenocarcinoma tissues and A-549 cells, indicated that quercetin could reduce mRNA and protein expression of Claudin-2 in A-549 cell lines without involving Akt and ERK1/2, whereas, Claudin-2 was declined by quercetin mediated by enhancement of miR-16, which is down-regulated in NSCLC, significantly (101). Inflammation and 20–40% of cancer cases are correlative and blocking of cytokines related to inflammation is defined as an adjunct therapy in combination with tumoricidal drugs (94). A common pollutant found in the atmosphere and recognized as a human carcinogen is Nickel. Some studies reported nickel-containing compounds as potential risk factors in lung cancers. Five phytochemicals such as quercetin, curcumin, chrysin, apigenin, and luteolin could suppress the effects of NiCl2 (Ni) on migration and invasion in lung cancer cells. In addition, those suppressed inductions of IL-1β, IL-6, TNF-α, and IL-10 with Ni. Furthermore, both quercetin and chrysin suppressed the expression of TLR4 and MYD88 and decreased phosphorylation of IKKβ and IκB, as well as the face of MMP-9 exposed to Ni (102). Also, the blocking of the cell cycle division was seen at the G2/M checkpoint (94). The recent scientific literature has also documented that after radiation-resistant GLC-82/R and HTB-182/R cell lines of NSCLC were treated with different doses of quercetin, downregulation of miR-16-5p and elevation of WEE1 (a tyrosine kinase that up-regulated in cancers and can modulate G2/M checkpoints before mitosis) mRNA expression, and survival ratios of cell lines were seen remarkably. Also, with knockdown WEE1, the survival of both radioresistant and wild-type cells (103). A range of studies has described that receptor tyrosine kinase (RTK) and cyclin-dependent kinase (CDK) were deregulated and enhanced noticeably in lung cancer. Some of the RTKs that were highly expressed in lung cancer cell lines are the Epidermal growth factor receptor (EGFR), fibroblast growth factor receptor 1 (FGFR1), insulin-like growth factor 1 receptor (IGF1R), and tyrosine-protein kinase Met (MET). Quercetin played a role in those, and the binding mechanism of quercetin inhibited RTKs. CDK6, which supports the growth and viability of various cancer cells, was hampered by the dose-dependent manner of quercetin (IC50 dose of QR for A-549 cells is 52.35 ± 2.44 μM). Moreover, the effect of quercetin-mediated inhibition of CDK6 caused of change in the colonization of A-549 cells and apoptosis induction, which was induced after 48h of treatment (16.22% in A-549 cell line) (104, 105). Hong Li et al. and colleagues reported that Yang-Yin-Qing-Fei-Tang (YYQFT) is a traditional Chinese medicine with multiple components that proved an anti-tumor in cancer therapy. One of the components of YYQFT is quercetin, which inhibited growth cells in the Lewis lung cancer (LLC) at 300 μM, 41% of cells compared with other components in YYQFT like catechin and gallic acid (13.3% and 18.8% of cells, respectively) (106).

### *In vivo* studies

Due to this apoptosis, survival, angiogenesis, inflammation, and cell cycle process, the observed noticeable effect of quercetin has been reported in various *in vivo* studies (107). A study observed that in Male laka mice, administration of both phytochemicals curcumin and quercetin combined with benzo (a) pyrene (BP) could modulate the post-translation of p53 related to apoptosis. BP-treatment mice improved their body weight growth with curcumin and quercetin separately and in combination. Further, tumor nodules, lung weight, and tumor incidence following treatments with curcumin and quercetin both separately and in combination were decreased markedly. In contrast, the assay depicted a significant increase in activities of caspase3 and 9 in lung cancer of mice (108). Also, immunoblot studies revealed quercetin, and curcumin-treated lung of mice significantly decreased BCL-2 expression and improved BAX protein expression in lung cancer cells. On the other hand, these results in the BP-treated mice were seen as vice versa. In addition, combination of BP +curcumin +quercetin brought a significant increase and activation of caspase-3/9 compared with BP-treated group (4.92 ± 0.12, 1.77 ± 0.15, and 4.00 ± 0.17, 1.42 ± 0.10, respectively). Also, the TUNEL assay depicted treatment with these supplements separately and in combination induced apoptosis and increased apoptotic cells compared with the BP-treated mice group (23.33 ± 2.08, 5 ± 1.0, respectively) (109). Moreover, Quercetin-loaded mixed micelles (Que-MMICs) were formed from 1,2-distearoyl-sn-glycerol-3-phosphoethanolamine–poly (ethylene glycol)–biotin (DSPE–PEG–biotin) and poly (ethylene glycol) methyl ether methacrylate–poly[2-(dimethylamino) ethyl acrylate]–polycaprolactone (PEGMA– PDMAEA–PCL) for NSCLC treatment in A-549 cells by Kangkang Li et al. this action resulted in efficient encapsulation of quercetin into mixed micelles until more than 85.7%. Cytotoxicity of Que-MICs and free Quercetin was improved by Que-MMICs, which efficiently induced apoptosis and arrested cell cycle in NSCLC and A549 tumor xenograft model established in nude BALB/c mice (110). From the mentioned literature, quercetin can act as a potential growth inhibitor of tumor cells (107). In cetuximab quercetin or paclitaxel nanoparticles (Cet-Que NP or Cet-PTX NP) group of BALB/c mice, inhibited tumor weight (111). With attention to the previous heading, the effects of quercetin binding to aurora B caused smaller size and growth of tumors in the xenograft mouse model (99). Similarly, after 15 days of YYQFT treatment in C57BL/6 mice, significant inhibition of growth tumor was detected related to high-dose quercetin. A high dose of quercetin (200μg/ml) enhanced inhibitory effects on tumor growth rather than the low dose (50μg/ml). Especially the size and the number of metastatic lesions were depicted as much smaller at 200μg/ml of quercetin (106). Quercetin also inhibited invasion in cell line cancers (107). Jer-Hwa Chan et al. demonstrated that SCID mice were exposed to A-549 cells and 50Mm quercetin. This quercetin-treated cell depicted decreased lung colony formation that revealed suppression of bone metastasis of NSCLC (33). In a similar article in 2018, stearoyl-L-a-phosphatidylethanolamine-polyethylene glycol 2000-RGD (a short peptide containing arginine-glycine-aspartic acid, which acts as a recognition site for integrins and their ligands and a target for angiogenesis) liposomes ([DSPE] -PEG2000-RGD-LPs/QCT was identified as an anti-tumor in C57BL/6 mice and rat with A-549 tumors (112).

## Potential therapeutic functions of quercetin and nanoparticles in lung cancer

Quercetin combined with delivery carriers like polymers, liposomes, chitosan, and other drug carriers enhanced solubility, absorption, circulation time, and target specificity (107). Utilization of mesoporous silica nanoparticulate like SBA-15 was defined as drug delivery purpose. Saad Alkahtani et al. revealed that 10μM quercetin-loaded SBA-15 (Que-SBA-15) decreased p-AKT, PI3K, and increased mTOR, caspase 9, and cytochrome c versus control samples (113). Cheng-Hung Chuang et al. demonstrated that trichostatin A (TSA), a histone deacetylase inhibitor, could not induce an apoptotic pathway in H1299 cells significantly until 72h. In contrast, quercetin, in combination with TSA, elevated apoptosis after 48 and 72h by about 62% and 88%. Also, death receptor 5 (DR5) levels, which is well known to mediate apoptosis pathways, mRNA, and protein, by 60% and 77%, respectively. Moreover, dual particles enhanced caspase3/10 and p300 expression. Studies have shown that quercetin itself, combined with chemotherapy, increased apoptosis in cancer cells (111). Furthermore, Yonghong Wang et al. reported an effective first-line chemotherapeutic drug, Paclitaxel (PTX), used for NSCLC treatment. Flow cytometry showed quercetin could increase cell cytotoxicity of PTX, and a combination of quercetin and PTX promoted apoptosis. Furthermore, Akt and ERK phosphorylation had no effective treatment with PTX, while that was markedly inhibited by the combination of quercetin and PTX (111). Similarly, Seung Hyun Lee and colleagues reported cell viability after being treated with quercetin and gemcitabine together, along with various concentrations of gemcitabine. A noticeable reduction was shown at 0.01 μg/ml dose of gemcitabine in both A-549 and H460 cell lines compared with gemcitabine alone (all p<0.05). further, a marked increase in caspase-3 activity at 0.1 μg/ml doses of dual molecules was observed, and a maximum increase of that was detected, 5.8-fold at 10 μg/ml and 9.2-fold at 1 μg/ml in A-549 and H460 cells, respectively. Also, quercetin combined with gemcitabine resulted in a downregulation of HSP70 expression significantly related to apoptosis (111). Liposomes consist of amphiphilic lipid molecules and can carry and transport hydrophilic and hydrophobic therapeutic agents (107). Muhammad Kashif Riaz et al. found that T7 was identified as a cell-targeting peptide with a specific binding affinity for TFR, which overexpressed on the surface of cancer cells, especially in the A-549 cell lines. Exposed quercetin to various types of T7 surface-functionalized liposomes (T7 targeted liposomes) along with its effect on lung cancer reported by Muhammad Kashif Riaz et al. they mentioned that on liposome surface in A-549 cells, T7-QR-Lip enhanced cytotoxicity, induction of apoptosis, and S-phase cell-cycle arrest by increasing T7 peptide, which increased the ability of liposomes to penetrate lung cancer cells by targeting TFR, density compared with free quercetin and non-targeted quercetin liposomes (QR-Lip). In addition, higher concentrations of T7-QR-Lip showed significant cytotoxicity (3 fold) compared to free QR. Further, T7-QR-Lip increased the anti-cancer activity of QR and lifespan of mice (p<0.01) (112). In a partly similar study, quercetin in combination with copper salt can improve solubility and suitable for intravenous administration in liposomal formulations. Analyses revealed that the area under the curve (AUC) for quercetin -copper in liposomes was 8382 μg h/ml, while for quercetin along with CuG (as a control) in liposomes was 1577 μg h/ml that, related to the rate of quercetin release into plasma and circulation of liposomes in mice (114). It is worthwhile to mention that exposure of A-549 cells to quercetin along with PMX, PMX/DCK showed concentration-dependent cytotoxicity with synergistic reduction of cell viability after treatment with 10, 20 μg/mL quercetin (61.0% ± 2.51% and 53.4% ± 4.42%, respectively), and suppressed transitions of cell-cycle at G2/M, G1/S, and cyclin-dependent kinases. Next, a wound-healing assay showed a higher sensitivity with reductions in cell proliferation/migration in cells treated with PMX/DCK and quercetin (30.5% and 14.9%, respectively). Also, PMX combined with quercetin (2.53-fold) showed a significant increase in the intestinal membrane permeability compared with free PMX. Further, quercetin was incorporated into NE, which resulted in higher permeability through the artificial membrane and Caco-2 cell Monolayer (16.6 ± 0.621 and 8.46 ± 2.21, respectively) compared with quercetin in water and a combination of 0.3% CMC. In addition, after oral administration of PMX/DCK-QCN-NE in rats, the Cmax and AUC last were increased compared with oral quercetin in 0.3% CMC (22.0- and 23.9-fold, respectively). The anti-cancer effect in A549 cell-bearing mice after once-daily oral administration of PMX/DCK-QCN-NE showed an inhibition and reduction of tumor growth and tumor mass compared with the oral PMX/DCK-NE and control groups (115). Moreover, cell proliferation of A-549 cells in lung cancer depended on quercetin -mediated gold nanoclusters (Que-GNCs) in a dose-dependent manner and a time-reliant manner from 0-24h. MMT assay showed the cell proliferation at 100 μg/ml concentration of Que-GNCs, which resulted in potential cytotoxicity from irregular cytoplasm with rupture in the cell membrane to complete damage in the cellular structure inhibited, and elevation of dead cells in that cell line was observed. In addition, using Que-GNCs measured by immunofluorescent probe induced enhanced reactive oxygen species (ROS) and oxidative stress at their doses from 50 μg/ml and even higher doses of 100 and 150 μg/ml (111). Summary of previous studies on the therapeutic effects of quercetin in lung cancer are listed in **Table 2**.

**Table 2:** The therapeutic effects of quercetin on lung cancer.

Model	Type of Quercetin (Que)	Cell/Animal model	Result	Que Dose	Ref
*in vitro*	Que	A-549	increased BCL2/BAX mediated apoptosis expression and inhibition of vimentin and N-cadherin (cytoskeletal proteins that function in migration).	10, 30, 60 μM	(95)
*in vitro*	Que	A-549	downregulate IL-6, STAT-3, Bcl2 activity, NF-κB expression, and upregulate the Annexin and the PI cell population through induction of mitochondrial-mediated apoptosis.	10 – 100 μM	(96)
*in vitro*	Que	A-549	enhanced TRAIL-induced cell death *via* autophagy flux activation. Decreased the p62 protein expression and enhanced caspase-3 cleavage.	20, 40, 80 μM	(97)
*in vitro*	quercetin -metabolite-enriched plasma (QP)	A-549	inhibited cell invasion, Matrix metalloproteinases (MMP-2) activity and migration in dose-dependent effects.	2 – 10 μM	(98)
*in vitro*	Que	A-549	Reduced mRNA expressions of urokinase plasminogen activator (uPA) and inhibited mRNA expression of CXCL-8, CXCR1, ß-catenin connection to migration and invasion of EMT in lung cancer cells.	20-40 μM	(116)
*in vitro*	Que	A-549, HCC827	inhibited the migration/invasion of non-small cell lung cancer (NSCLC) cell lines through suppressing the EMT.	10–50 μM	(33)
*In vitro*	Que	A-549	Inhibited tumorigenesis in human lung cancer partially mediated *via* the elevation of miR-16 and decrease in claudin-2 expression without involving Akt and ERK1/2.	0, 0.5, 5, 50, 100 μM	(101)
*In vitro*	Que	GLC-82/R, HTB-182/R	Suppressed the viability and enhanced the radiosensitivity of NSCLC cells *via* increasing miR-16-5p expression and suppressing WEE1 mRNA expression in a dose- and-time-dependent manner.	50 μM	(103)
*In vitro*	Que	A-549	inhibited RTKs. CDK6, which supports the growth and viability of various cancer cells	52.35 ± 2.44 μM	(104)
*In vitro* + *in silico*	Que	MCF-7, A549	induced apoptosis, by decreasing the production of reactive oxygen species and CDK6 expression.	0–250 μM	(105)
*In vivo*	Que + curcumin + benzo(a) pyrene (BP)	Male laka mice	enhanced chemopreventive potential against development of lung carcinogenesis by stimulating the apoptotic machinery. decreased BCL-2 expression and improved BAX protein expression in lung cancer cells.	40 mg/kg	(109)
*In vitro* *+ in vivo*	Que-loaded mixed micelles (Que-MMICs)	nude BALB/c mice	induced apoptosis and arrested cell cycle in NSCLC and A549 tumor xenograft model established.	0-50 μg/ml	(110)
*In vitro* *+ in vivo*	YYQFT	C57BL/6 mice	inhibited growth tumor related to high-dose quercetin. decreased BCL-2 expression and improved p53, Fas, Bax and Bag expression in lung cancer cells.	200 μg/ml	(106)
*In vitro* *+ in vivo*	Que + SBA-15	A-549	Induced cell death *via* PI3K/AKT/mTOR signaling pathway and increased mTOR, caspase 9, and cytochrome c	0, 20, 40, 80, 100 μg/ml	(117)
*In vitro*	Que+ TSA	A-549, HCC827	inhibited the migration/invasion of NSCLC cells through suppressing the EMT. suppressed snail-dependent Akt activation by upregulating maspin.	1–100 μM	(111)
*In vitro*	Que + PTX	A-549, A-549/Taxol	Combination of quercetin and PTX suppressed cell growth and induced apoptosis. Que reversed PTX resistance of lung cancer cells by inhibiting the phosphorylation of Akt and ERK.	0, 5, 10, 20, 40, 80 μg/ml	(118)
*In vitro*	Que + gemcitabine	A-549, H460	Reduced cell viability and suppressed HSP70 expression in both cell lines dose-dependently.	10, 50, 100, 200 μM	(119)
*In vitro* *+ in vivo*	Que + PMX	A-549	showed concentration-dependent synergistic inhibitory effects on cancer cell proliferation/migration.	20 µg/mL	(115)

Note: Mesoporous silica nanoparticles (SBA-15); Trichostatin A (TSA); (Polymeric microspheres, PMs), PMs loaded with paclitaxel (PTX); Pemetrexed (PMX).

## Prostate cancer

One of the most common cancers diagnosed in adult men is prostate cancer (PCA) (120), which is classified as malignant with faster-growing cells and benign with slow-growing cells (121). After lung cancer, PCA is the second leading reason for cancer-related death in the United States and impacts about 70% of patients over 65 worldwide (8). The therapeutic influences of quercetin in different cancers, including prostate cancer, have been evaluated through *in vitro* and *in vivo* trials (55, 122–124). So, it may prevent the onset of this type of cancer because it indirectly blocks the promoters of two essential genes in the pathogenesis of prostate cancer, including androgen receptor (AR) and prostate-specific antigen (PSA) (125).

### *In vitro* studies

Several laboratory studies have shown the cytotoxic effects of quercetin on prostate cancer cells compared with normal prostate cells. As mentioned earlier, this material is involved in inhibiting AR function, secretion of androgen-regulated tumor biomarkers, PSA antigen, and hK2, and inhibiting PSA, NKX3.1, and ODC transcript levels. In other words, quercetin has a role in preventing or treating prostate cancer by repressing AR expression (125).

According to the statement made in Al-Jabban’s research, several previous studies have shown that quercetin has been able to repress the growth of androgen-sensitive (LNCaP) and androgen-resistant human prostate cancer cell lines (PC-3 and DU145) while having no noticeable toxicity to normal prostate epithelial cells (126). One group of researchers stated that LNCaP and PC-3 cells with oxidative cellular environment turned off ROS after quercetin treatment. At the same time, DU-145 showed an increase in ROS levels despite a very reducing environment. Furthermore, a contrasting effect of quercetin on the PCa cells survivorship pathway was also observed. PCA cells with p53 mutated (DU145) and elevated ROS show a noticeable lessening in activation of the Akt track promoting survival, whereas Raf/MEK was activated in reaction to this medication. The p53 and PTEN-free PC3 cells with reduced ROS levels exhibited considerable activation of the Akt and NFκB signaling trajectory (127). They proved that treatment with quercetin dramatically diminished PCa cell viability (LNCaP, DU-145, and PC-3) over time and dose. The outcomes of the apoptosis assay indicated that the highest apoptosis was observed in LNCaP, followed by PC-3 and DU-145 cells after three days and nights of quercetin treatment. Also, the upshots point to the induction of apoptosis by quercetin in PCA cells followed by secondary necrosis over a while. According to previous studies, a significant indicator of cancer cells escapes apoptosis (128), while quercetin inhibits this escape in PCA cells by regulating apoptotic pathway components. In other words, androgen-sensitive LNCaP cells with wild-type p53 showed a decrease in BAX and BIM expression at all three-time points and a reduction in PUMA expression at 24 h, followed by subsequent times. In DU-145, androgenic-independent cells with mutant p53, the treatment with quercetin showed decreased BAX levels for 24 hours and increased over 48 and 72 hours. On the other hand, BIM significantly reduced after two days.

Nevertheless, PUMA has been shown to decrease significantly after one day. PC-3 cells, androgen-independent cells lacking p53, showed a significant decrease in BAX and BIM expression overnight and increased PUMA expression for two days and nights. So, in their result, LNCaP and PC3 cells with the oxidized cell environment showed a ROS turning off after quercetin treatment, whereas DU145 illustrated increased levels of ROS despite the highly reducing environment. The opposite effect of quercetin on the PCA cells survivorship pathway was also observed. DU145, PCA cells with p53 mutated, and augmented ROS in reaction to quercetin showed a noticeable deduction in Akt’s pro-survival trial activation. In contrast, Raf/MEK was activated in response to this medication. The p53 and PTEN-free PC3 cells with reduced ROS levels exhibited considerable activation of the Akt and NFκB signaling trajectory (127).

Over the last few years, numerous researchers have expressed interest in using quercetin as novel anti-cancer therapy to inhibit angiogenesis and tumor growth (129, 130). Feiya Yang et al. also reported that following treatment with various amounts of quercetin over some time, the proliferation of PC-3 cells was stopped in a dose-dependent form. In that study, the effect of quercetin inhibition on PC-3 cell migration was investigated. It was found that this drug in different concentrations for 24 hours increased the rate of inhibition and invasion of these cancerous cells. In other words, there is a positive relationship between the degree of inhibition of this drug and increasing its dose (131). Thrombospondin-1 (TSP-1) is an anti-angiogenic agent that can inhibit angiogenesis and tumorigenesis (132). The investigation mentioned above reported the role of quercetin inhibition of TSP-1 angiogenesis in prostate cancer. According to their findings, after treating PC-3 cells with quercetin for 24 hours, the expression of TSP-1 mRNA and its protein was significantly increased relative to the control. In addition, increasing the quercetin dose was associated with increased TSP1-mRNA and protein expression (131).

Quercetin nanoparticles (QN) have been reported to be effective in treating malignant tumors (133). Cell exposure to QN can potentially induce cell death, particularly in cancerous cells (134). In one study, QN was used to remove the PCA *via* the hedgehog (Hh) signaling path. In average growth, this pathway controls tissue polarity, cellular proliferation, and cellular differentiation signaling. Anomalous signaling in this track has been reported in various human cancers, including prostate cancer (135–138).

Another group of researchers demonstrated the inhibitory role of QN in the development of PCA in the LNCaP cell lineage by enhancing the Hh signaling pathway. However, the effects of apoptosis and the anti-proliferative molecular mechanism of QN have not been investigated (135). The significant causes leading to the invasive and metastatic potential of cancer cells are increased expression of N-cadherin and decreased E-cadherin, which are a characteristic associated with epithelial cancers. In addition, various researchers have claimed that a possible drug target in cancer treatment is N-cadherin. Also, for exploring the epidermal growth factor receptor (EGFR) in hormone-resistant prostate cancer, PC-3 cells with no susceptibility to androgen are a perfect model. EGFR has a meaningful function in metastasis and tumorigenesis in various human cancers. A panel of scientists studied the effect of quercetin on the epithelial-mesenchymal (EMT) transition triggered by EGF in PC-3. They concluded that quercetin reverses EGF-induced cadherin switching by lowering EGF-induced EMT tags through EGFR/PI3K/Akt route, inhibiting EGF-induced infiltration and migration of PC-3 cells (139).

Q-6-C-β-D-glucopyranoside (QCG) is more stable compared to quercetin. In this regard, researchers have studied the effects of QCG on PC-3, LNCaP, and DU-145 as prostate cancer cells and RWPE-1 as a common cell lineage. Experiments have shown that QCG inhibits prostate cancer cell proliferation by inhibiting the cell cycle in the G0/G1 phase and induces apoptosis by releasing cytochrome c, caspase-3, and poly ADP-ribose polymerase. QCG also inhibited the production of ROS and Akt/mTOR trail. It showed anti-cancer activity against PC-3 cells by inhibiting the Akt-mTOR pathway through the aryl hydrocarbon receptor (140).

Another group looked at the antitumor effects of quercetin on several lines of prostate cancer cells, such as LNCaP cells and PC3 cells. They stated that quercetin inhibited the growth of both LNCaP and PC-3 cancerous cells through apoptosis (55).

Other scientists have also studied the effects and mechanisms by which quercetin exerts its antitumor function against prostate cancer. They had measured multiple factors such as PI3K, AKT, P53, MMP-2, and MMP-9 that have an essential role in biological activities, including proliferation, apoptosis, proliferation, cell cycle, migration, and invasion in the DU-145 and PC-3 cell lines. In general, quercetin had inhibited dose-dependently some biological activities such as cell proliferation, migration, and invasion in mentioned cell lines. In other words, quercetin up-regulated the rates of G1 phase cell cycle and cellular apoptotic in both examined cell lines compared with the control group, while it declined the expressions of the PI3K, AKT, MMP-2, and MMP-9 proteins (141).

### *In vivo* studies

One study demonstrated the anti-tumor function of quercetin in a sample of an androgen-sensitive LAPC-4 xenograft prostate tumor utilizing severe combined immunodeficiency (SCID) mice (142). It can also decline the volume and poundage of PC-3 tumors in 6-week-old BALB/c A nude mice by targeting the VEGF-R2 controlled AKT/mTOR/P70S6K signaling path (17). Also, another study showed that at the end of a 4-week intervention in nude male BALB/c mice, the PC-3 tumors in the quercetin -treated groups were more undersized than controls. Even at high doses of the drug throughout the treatment process, no drug-related toxic reactions or side effects such as poor mood, seizures, and hematuria were reported in mice. Weight loss in the treated groups did not significantly differ from the control group. Quercetin impedes microvascular tumor density in xenografted tumor tissues. Also, tests showed CD31- and CD34-positive vessels in PC-3 xenograft transplant tumor tissue. Immunohistochemical tests showed CD31 and CD34 expression on vessels in PC-3 xenografted transplant tissue, confirming quercetin ‘s ability to inhibit angiogenesis in this type of cancer cell transplant in a dose-dependent manner. This study also showed that quercetin increased TSP-1 mRNA and protein expression to inhibit angiogenesis, thereby inhibiting the growth of human prostate cancer *in vivo* (131) (**Figure 2**).

Su Z et al., similar to their performed experiments *in vitro*, evaluated the effects and mechanisms by which quercetin inhibits a range of biological activities *in vivo* on nude mice infected with prostate tumors. They obtained similar results to their *in vitro* outcomes mentioned above. In summary, quercetin exhibited suppressive functions in tumor proliferation, invasion, and migration and an increase in apoptosis and G1 phase rate (141).

## Combination of quercetin with other drugs in prostate cancer

Resveratrol has previously been shown to block key signaling tracks associated with tumor onset and progression (143–145). The pro-apoptotic effects of resveratrol on human PCA cells have been demonstrated. On the other hand, its antiproliferative effects against PCA cells may be mediated by modulation of the PI3K/AKT path and the BCL2 proteins group (146). Harper et al. showed that resveratrol suppresses the progression of PCA in a TRAMP (transgenic adenocarcinoma of the mouse prostate) model (147). Another group found that resveratrol suppresses PCa growth and induces apoptosis in the TRAP (Transgenic Rat of Prostate Adenocarcinoma) model (148). As mentioned, quercetin, like resveratrol, can inhibit PCA cells (131, 149, 150). To this end, Chandra K. Singh and colleagues stated that the combination of Que-resveratrol has notable anti-tumor effects on a PCA mouse sample because their preclinical data showed a considerable lessening in tumor size and weight group (131). Ki67 and proliferating cell nuclear antigen (PCNA) are biomarkers that have important roles in cell proliferation, rapid growth, and cell division. 4-hydroxynonenal (4HNE) is a biomarker of oxidative stress, while Survivin prevents programmed cell death (151). They found significant reductions in 4HNE in all three treatment groups, which may be due to the reduction of 4HNE oxidative damage in PCA. They also observed significant reductions in Ki67 and PCNA proliferation markers and cell survival markers in response to quercetin and/or resveratrol.

In all three treatment groups, compared to the control, there was a significant increase in the incision of both caspase-8 and -9, which indicates the involvement of external and internal apoptotic trails in the inhibition of this type of tumor. In addition, a significant decrease in BCL2 was observed in all treatment groups and with a further reduction in the group with the quercetin -resveratrol combined treatment. On the other hand, no significant difference in BAX was shown either at the protein or mRNA levels. Their results indicated that the blend of quercetin and resveratrol might regulate some essential genes and signaling pathways that are dysregulated in PCA and thus may help treat PCA (143).

2-Methoxy estradiol (2-ME), a biologically endogenous product of 17β-estradiol (E2), has been shown to play an anti-tumor role in many tumors (152). In prostate cancer, 2ME can inhibit both androgen-dependent androgen-independent cancer cells *in vitro* and *in vivo*. Nonetheless, 2ME has the disadvantage of limited biological access and rapid degradation. A combination of drugs with fewer side effects and higher anticancer effects than monotherapy was proposed (153–156). Guodong Wang investigated quercetin’s antiproliferative and apoptotic activity in combination with 2ME in both androgen-dependent LNCaP and androgen-independent PC3 prostate cancer cell lines. They showed synergistic antiproliferative and pro-apoptotic activity that raised the G2/M stage cell population and significantly reduced the Bcl2/BAX ratio. The mixture of quercetin and 2ME is a new clinically pertinent therapy regimen that may enhance the anti-tumor influence on prostate cancer and reduce the side effects of quercetin or 2ME alone (157). These researchers also treated nude male BALB/c mice with PC3 and LNCaP cell xenograft tumors *via* quercetin or 2ME and their blend for one month. They observed that PC3 and LNCaP xenograft transplant tumors were more petite in the quercetin or 2ME cure group than in the control. Their suppression was more noticeable in the quercetin and 2ME mixture groups due to enhanced induction of apoptosis, enhanced inhibition of the phosphatidylinositol 3 kinase (PI3K)/Akt path, and angiogenesis (158). Another group assessed the synergistic impact of quercetin and 2ME on PC3 cells. The data demonstrated that both quercetin and 2ME could impede PC3 cell proliferation time-dependent. Synergistically, these agents showed higher antiproliferative effects. The potential for PC3 cell migration after drug treatment was also evaluated separately and synergistically. The blended group has a more substantial anti-migratory effect than each drug group and emphasizes the interaction between quercetin and 2ME in exerting anti-tumor effects against PCA metastasis (159). One study examined the influences of quercetin and vitamin C therapy on factors that influence the growth, metastasis, and angiogenesis of prostate cancer. Their results showed that treating prostate cancer cells with quercetin or vitamin C lessened the expression of chemokine receptors CXCR4 and CXCR7, but their blend therapy had a better influence. In addition, their results showed that quercetin and vitamin C reduced the expression of integrin α4,α5, and β1, which have an essential role in the adhesion and metastasis of these cancer cells in DU145 PC3 cells. Therefore, the use of this drug combination has shown an increase in quercetin efficiency in treating prostate cancer (160).

Several researchers have studied the anticancer power of the combination of arctigenin (Arc) and quercetin in prostate cancer. Because Arc is a potent inhibitor of AR signaling in LAPC-4 prostate cancer cells, the combined effects of Arc and quercetin on proliferation, apoptosis, cell migration, and underlying mechanisms in both androgen-dependent prostate cancer cell lines (LNCaP and LAPC-4) have been reviewed. Finally, their results showed that the combination therapy of Arc and quercetin increased the inhibitory effect of this drug on the growth of androgen-dependent prostate cancer cells, which was associated with increased regulation of several signaling pathways, including the AR and PI3K/Akt pathways (161).

Interestingly, one study used a synthetic analog of fistin and quercetin flavanols called TMFol (3 ‘, 4’, 5’-trimethoxyflavonol) to evaluate the therapeutic effect of this substance in prostate cancer. This investigation assessed the impact of TMFol in some prostate cancer cell lines, including 22Rv1, TRAMP C2, PC-3, and LNCaP, and in nude mice with 22Rv1 or TRAMP-C2 tumors. They showed that TMFol inhibited cell growth in all explored prostate cancer cell lines more powerfully than fistin or quercetin alone. It also inhibited the growth of TRAMP C2 tumors *in vivo*, whereas either fistin and quercetin alone lacked such activity in this model. In addition, TMFol reduced the development of the 22Rv1 tumor *in vivo*. This group of researchers knew that the main reason for the effectiveness of this drug in both models was apoptosis. *In vitro*, however, apoptosis was induced only in TRAMP C2 cells affected by this drug (162).

Due to the expanded ability to proliferate, migrate, and advance the expression and secretion of the metalloproteinase 9 (MMP9) matrix, the Du145III cells have a high invasive capacity. So that Tsai PH et al. evaluated the influence of dietary flavonoids such as luteolin and quercetin on the invasive power of Du145III cells by activating the JNK pathway. They showed that these medicines suppressed the expression of highly invasive Du145III cell malignancies, vasculogenic mimicry (VM), the formation of anchorage-independent spheres, and specific cancer stem cell (CSC) markers. Also, because they targeted CSC cells and prevented the invasion of cancer cells, maybe there is an anti-angiogenic and anti-metastatic role in these compounds (163).

Another study evaluated the synergistic effect of metformin with quercetin on prostate cancer cells. It confirmed co-treat effects on inhibiting the growth, migration, and invasion of the PC-3 and LNCaP cells. In the combined treatment of these factors, they observed more apoptosis than in the one-factor treatment induced by caspase and Bcl-2 family components. They also documented that the use of this combination dramatically impeded the VEGF/Akt/PI3K path. *In vivo* in nude mice showed a synergistic effect of these compounds in inhibiting the growth of prostate cancer cells (164).

A study investigated combining quercetin with curcumin on demyelination and AR re-expression in CaP cell lines -free AR such as PC3 and DU145. Finally, it has been documented that the combination of these two drugs has a significant effect on DMNT control, associated with hypomethylation, recovery of mRNA and AR protein levels, and induction of apoptosis through mitochondrial depolarization of PC3 and DU145. The result was re-sensitization of androgen-resistant CaP cells to AR-mediated apoptosis (165).

It has previously been suggested that green tea and quercetin target cellular signaling pathways from onset to tumor progression (166, 167). Also, docetaxel (Doc) can be an ideal drug for treating metastatic and castration-resistant prostate cancer. In the research of Piwen Wang et al., the role of green tea and quercetin in sensitizing prostate cancer cells to Doc has been investigated *in vitro*, including LAPC-4-AI and PC-3. According to the researchers, the increase in a stoppage in the G2/M phase and the increase in apoptosis in LAPC-4-AI and PC-3 cells compared to Doc alone due to the use of drug combinations including quercetin, and Doc, epigallocatechin gallate (EGCG) had observed. Overall, these agents increased PI3K/Akt inhibition and signaling and transcriptional activator (Stat) 3 compared to Doc alone while reducing the expression of the multidrug-resistant protein (MRP1). In addition, combining EGCG and quercetin compared with Doc alone showed an increase in tumor cell permeability and cell proliferation inhibition in LAPC4AI and PC3 cells (168).

## Quercetin in patients with refractory prostate cancer

Over the ex-two decades, many advances in PCA screening, treatment, and monitoring have greatly improved the quality of life and survival after diagnosis. However, more than 20% of patients diagnosed with PCA are still resistant to the treatment (169).

Docetaxel is the first-line chemotherapeutic agent for metastatic prostate cancer. Nonetheless, the emergence of resistance diminishes its effectiveness and restrains its survival benefits. An investigator group documented that quercetin inverts docetaxel resistance in the docetaxel-resistant cells (LNCaP/R, PC3/R) on proliferation, colonization, migration, infiltration, and apoptosis functions. In other words, they found that combination therapy of these two drugs significantly blocked the PI3K/Akt trail and boosted apoptosis (170).

While doxorubicin is used to treat prostate cancer, there is a high rate of chemotherapy failure due to the development of prostate cancer cells’ resistance to doxorubicin. A group suggested that c-met is associated with drug resistance in prostate cancer. Furthermore, quercetin treatment was shown to inhibit c-met expression in PC3/R cells significantly. Subsequent inhibition of the PI3K/AKT signaling pathway downstream of c-met was also observed. In addition, combination treatment with quercetin inhibited the PI3K/AKT pathway, resulting in doxorubicin-induced mitochondrial dysfunction, ROS release, caspase 3/9 cleavage, and apoptosis in PC3/R cells. Overall, it has been shown that combination therapy with quercetin can invert doxorubicin resistance in prostate cancer cells by targeting the c-met/PI3K/AKT ways (171).

Paclitaxel (PTX) is a chemotherapeutic drug used to treat cancer, including prostate. PTX causes apoptosis by disrupting the dynamic equilibrium between soluble tubulin dimer and polymerized tubulin, inhibiting metaphase-to-late cell migration (172). What is more, several clinical studies have confirmed that PTX increases survival in these patients. On the other hand, PTX has many side influences and can cause acquired drug resistance after treatment, thereby hindering its clinical application as an anticancer drug (173). In this regard, a group of scientists mixed Quercetin and PTX to treat prostate cancer and assessed their anti-tumor effects *in vitro* and *in vivo*. *In vitro* studies explored cancer cell proliferation, apoptosis, cell cycle arrest, and ROS production after treating them with Quercetin and PTX. They also examined intracellular endoplasmic reticulum (ER) stress and the ability to migrate. They also analyzed the effects of this mixture therapy on PC3 mice with cancer and evaluated possible anticancer mechanisms by immunohistochemical staining of several related proteins. They also examined the impact of the mixture of these drugs on PC3 mice with cancer. They discovered that Que+PTX synergistically inhibits the growth of prostate cancer cells, induces cell cycle arrest in the G2/M phase, and induces cell apoptosis. Also, quercetin effectively causes intracellular ROS production and ER stress, and downregulation of hnRNPA1 may underlie the inhibition of cell migration (89).

Due to its poor water solubility and low bioavailability, the clinical use of quercetin has been impaired. A group of researchers investigated the therapeutic effect of new nano micelles loaded with QCT (M-QCTs) on refractory prostate cancer. They found that QCT was effectively encapsulated in micelles, increasing the drug’s solubility in water. However, their study showed that the semi-maximal inhibitory concentration value of M-QCT was much lower than that of free QCT. Therefore, MQCT was significantly more potent than free QCT in inhibiting the proliferation of human androgen-independent PC3 cells and inducing apoptosis. In addition, M-QCT showed a perfect anti-tumor efficacy and almost a 50% reduction in tumor growth rate in the PC3 xenograft mouse model. This may be due to increased MQCT accumulation at the tumor site due to improved permeability and retention (EPR). Their study showed that MQCT remarkably increased drug assembly at the tumor site and had excellent anti-tumor activity in this cancer. Therefore, this novel drug delivery system represents a suitable and practical therapeutic technique for clinical therapy (174). Summary of previous studies on the therapeutic effects of quercetin in prostate cancer are listed in **Table 3**.

**Table 3:** The potential therapeutic effects of quercetin on prostate cancer.

Model	Type of Quercetin (Que)	Cell/Animal model	Result	Que Dose	Ref
*In vitro*	Que	LNCaP, DU-145, PC-3	Induces cell death in malignant cells and simultaneously decreases cell survival in PCa cells. LNCaP and PC-3 cells with oxidative cellular environment turned off ROS after quercetin treatment. At the same time, DU-145 showed an increase in ROS levels despite a very reducing environment	5, 10, 20, 40, 80, 160 μM	(127)
*In vitro*	Que + doxorubicin	PC3	Reversed the doxorubicin resistance of prostate cancer cells by inhibiting c-met expression in PC3/R cells. promoted the doxorubicin-induced apoptosis in PC3/R cells through the mitochondrial/reaction oxygen species pathway.	10 µM	(171)
*In vitro + in vivo*	Que	PC-3, HUVECs	increased TSP-1 expression to inhibit angiogenesis resulting in antagonizing prostate cancer PC-3 cell and xenograft tumor growth.	0, 25, 50, 75, 100 μM	(131)
*In vitro*	Q-6-C-β-D-glucopyranoside (QCG)	LNCaP, PC-3, DU145, RWPE-1, HEK-293	induced a significant dose-dependent decrease in the cell viability. inhibited prostate cancer cell proliferation by inhibiting the cell cycle in the G0/G1 phase. induced apoptosis by releasing cytochrome c, caspase-3, and poly ADP-ribose polymerase.	1, 20, 40, 800, 100, 200 μM	(140)
*In vitro + in vivo*	Que	LNCaP, CT-26, MOLT-4, Raji, MCF-7	Up-regulated the rates of G1 phase cell cycle and cellular apoptotic in both examined cell lines. decreased the expressions of the PI3K, AKT, MMP-2, and MMP-9 proteins	50, 100, 200 mg/kg	(141)
*In vivo*	Que + resveratrol	PC3	Observed significant reductions in Ki67 and PCNA proliferation markers and cell survival markers. decreased the expressions of the BCL2 and the incision of both caspase-8 and -9	10 mg/kg	(143)
*In vitro*	Que + 2ME	LNCaP, PC3	showed synergistic antiproliferative and pro-apoptotic activity that raised the G2/M stage cell population and significantly reduced the Bcl2/BAX ratio.	1, 10, 100 μM	(157)
*In vivo*	Que + 2ME	nude male BALB/c mice xenograft	Enhanced induction of apoptosis, enhanced inhibition of the phosphatidylinositol 3 kinase (PI3K)/Akt path, and angiogenesis	75mg/kg	(158)
*In vitro*	Que + 2ME	PC3	synergistically downregulated mesenchymal markers with simultaneous upregulation of epithelial markers. downregulated the expressions of Wnt signaling proteins.	20 μM	(159)
*In vitro*	Que + vitamin C	DU145, PC3	lessened the expression of chemokine receptors CXCR4 and CXCR7. reduced the expression of integrin α4, α5, and β1	75µM	(160)
*In vitro*	Que + arctigenin	LNCaP, LAPC-4	increased the inhibitory effect on the growth of androgen-dependent prostate cancer cells, which was associated with increased regulation of several signaling pathways, including the AR and PI3K/Akt pathways	5, 10, 20 µM	(161)
*In vitro + in vivo*	Que + TMFol + fistin	22Rv1, TRAMP C2, PC-3, LNCaP	inhibited cell growth in all explored prostate cancer cell lines more powerfully than fistin or Que alone and inhibited the growth of TRAMP C2 tumors *in vivo*.	10 μM	(162)
*In vitro*	Que + luteolin	Du145III	suppressed the expression of highly invasive Du145III cell malignancies, vasculogenic mimicry (VM), the formation of anchorage-independent spheres, and specific cancer stem cell (CSC) markers	20 μM	(163)
*In vitro+ in vivo*	Que + metformin	LNCaP, PC-3	Exerted synergistic antitumor effects in prostate cancers *via* inhibition of VEGF/Akt/PI3K pathway.	10–40 μM	(164)
*In vitro*	Que + curcumin	DU145, PC3	Reduced DNMT activity and AR gene methylation, increased AR mRNA and protein.	0, 20, 40, 60, 80, 100 μM	(165)
*In vitro*	Que + Docetaxel	LNCaP/R, PC3/R	reversed docetaxel resistance in prostate cancer *via* androgen receptor and PI3K/Akt signaling pathways	5, 10, 20, 50, 100, 150, 200 μM	(170)
*In vitro+ in vivo*	Que + Paclitaxel	PC3 mice	Inhibited cell proliferation, increased apoptosis, arrested the cell cycle at the G2/M phase, inhibited cell migration, dramatically induced ER stress to occur, and increased ROS generation	7.5, 15, 30, 60, 120 μM	(89)
*In vitro+ in vivo*	nano micelles loaded with QCT (M-QCTs)	PC3 PC3 xenograft mouse model	enhanced antiproliferation and apoptosis induction ability in human androgen-independent prostate cancer models.	1, 10, 100 μM	(174)
*In vitro*	Que + arctigenin	LAPC-4, LNCaP	Enhanced the inhibition of cell migration in both cell lines and inhibited the expression of several oncogenic microRNAs including miR-21, miR-19b, and miR-148a.	10μM	(161)

2-Methoxyestradiol (2-ME).

## Conclusion

Quercetin constitutes the major bioflavonoid in the human diet. It exerts anti-inflammatory and chemopreventive effects. In addition, quercetin can improve cancer progression through various mechanisms including down regulation of mutant p53 proteins; G1 phase arrest; tyrosine kinase inhibition; and down regulation of cell survival, proliferative and anti-apoptotic proteins. The concept of quercetin as an anti-cancer compound is supported by preclinical studies. However, clinical studies that support these application are few and the results are mixed.

Limitations and suggestions:

1. Because of its antioxidant effects, quercetin may interfere with the actions of certain chemotherapy drugs.
2. Despite quercetin promising features for cancer therapy, low solubility, poor permeability, and short biological half-life time significantly confine its application in cancer therapy.
3. The pharmacologic response of other drugs can be markedly influenced by the concurrent administration of quercetin and its metabolites.

## Author contributions 

NL, ZY, MG, PK, BGh, MM, MhSh, PZB, and NE interpreted, and provided major contributions in writing the manuscript. All authors contributed to the article and approved the submitted version.
